# Elevated fruit nitrogen impairs oil biosynthesis in olive (*Olea europaea L*.)

**DOI:** 10.3389/fpls.2023.1180391

**Published:** 2023-06-30

**Authors:** Ran Erel, Uri Yermiyahu, Hagai Yasuor, Alon Ben-Gal, Isaac Zipori, Arnon Dag

**Affiliations:** Gilat Research Center, Agricultural Research Organization – Volcani Institute, Gilat, Israel

**Keywords:** fruit nitrogen, olive oil, oil-protein competition, over-fertilization, fruit-load.

## Abstract

Oil in fruits and seeds is an important source of calories and essential fatty acids for humans. This specifically holds true for olive oil, which is appreciated for its superior nutritional value. Most olive orchards are cultivated to produce oil, which are the outcome of fruit yield and oil content. Little information is available on the effect of nitrogen (N) on olive fruit oil content. The response of olive trees to different rates of N was therefore studied in soilless culture (3 years) and commercial field (6 years) experiments. In both experiments, fruit N level and oil biosynthesis were negatively associated. Fruit N increased in response to N fertilization level and was inversely related to fruit load. The negative correlation between fruit N and oil content was more pronounced under high fruit load, indicating sink limitation for carbon. These results agree with those reported for oilseed crops for which a trade-off between oil and protein was proposed as the governing mechanism for the negative response to elevated N levels. Our results suggest that the protein/oil trade-off paradigm cannot explain the noticeable decrease in oil biosynthesis in olives, indicating that additional mechanisms are involved in N-induced inhibition of oil production. This inhibition was not related to the soluble carbohydrate levels in the fruit, which were comparable regardless of N level. These results emphasize the importance of balanced N nutrition in oil-olive cultivation to optimize production with oil content.

## 1 Introduction

Nitrogen is a major essential plant nutrient and the most applied mineral in fertilization programs of horticultural crops (Kirkby, 2012) including olives (López-Villalta and Muñoz-Cobo, 1996; Therios, 2009). The preferred form in which N is taken up depends on soil conditions and plant species. In general, plants grown in alkaline soils, typical in traditional olive cultivation areas, predominantly take up N as $\text{N}{\text{O}}_{3}^{-}$ (Maathuis, 2009). Balanced N management in orchards is requisite for the optimization of olive productivity (Fernández-Escobar et al., 2012) and oil quality (Fernández-Escobar et al., 2006; Dag et al., 2009).

To optimize olive tree N nutrition, N status is commonly diagnosed by analysis of leaf N during mid-summer (July in the northern hemisphere) with concentrations below 1.3–1.4% of dry weight considered deficient (Fernández-Escobar et al., 2009b; Zipori et al., 2020). The importance of N fertilization for olive productivity was supported by several studies (Hartmann and Brown, 1953; Ferreira et al., 1986; Morales-Sillero et al., 2009; Haberman et al., 2019) who reported gradual increase in productivity in response to N application. Both excessive and deficient N levels significantly impair oil productivity (Erel et al., 2013a; Haberman et al., 2019), while high fruit N level is associated with decreased antioxidants concentration and overall oil stability (Erel et al., 2013a). In traditional agrosystems there is a debate regarding the necessity of annual N application for olive productivity as part of fertilization management since N is generally supplied in excess (Fernández-Escobar, 2011; Fernández-Escobar et al., 2012; Centeno et al., 2017). Additionally, N over-fertilization essentially leads to massive nitrate leaching below the root zoon (Segal et al., 2011; Erel et al., 2019) leading to the degradation of deep soils and ground water (Wick et al., 2012).

Olives are cultivated for their edible oil and fruit with roughly 90% of the world olive orchards (over 10 million ha) cultivated for oil production (FAOSTAT, 2020). Olive fruit is a drupe, composed of an external exocarp, a fleshy mesocarp and a woody endocarp surrounding the seed. The mesocarp is the largest tissue as it accounts for 60-70% of fruit dry weight. The mesocarp is the tissue of economic value because up to 98% of the oil accumulates there (Bellincontro et al., 2013). Oil synthesis and olive oil accumulation in the mesocarp cells of the fruit are influenced by many factors such as cultivar, fruit load, and environmental conditions. Fruit oil accumulation starts in early fruit growth stages, increases during the summer, being more intense between the pit hardening phase and the beginning of fruit maturation, to continue until fruit ripening towards the beginning of winter, when it levels off (Dag et al., 2011; Bellincontro et al., 2013; Dag et al., 2014; Bodoira et al., 2015). The dynamics of fruit oil accumulation are largely dependent of carbon supply by the leaves (Lavee and Wodner, 1991), but little is known regarding potential interaction with nutrient availability.

Fatty acid biosynthesis is vital for normal plant functioning and development. Fatty acids are integral components of cell phospholipids and other essential structures. This is specifically important in seeds and fruits where storage lipids represent an important source of calories and essential fatty acids for humans (Weselake, 2020). Despite numerous studies related to olive N fertilization, the effect of N nutritional level on oil biosynthesis has not yet been studied thoroughly. Some authors showed (but did not discuss) occasional decrease in oil content as a function of increasing N dose (Morales-Sillero et al., 2007; Fernández-Escobar et al., 2009a) while others showed no interaction (Centeno et al., 2017). In oilseeds, N fertilization has frequently been associated with decreased oil content as widely discussed in a review by Rathke et al. (2006) for rapeseeds, and recently also shown in sesame (Golan et al., 2022). In a meta-analysis study in the oilseed crop, covering 128 field trials, Si et al. (2022), indicated that N fertilization level significantly increased seed protein content while decreasing oil content. The common paradigm claims that competition for carbohydrates between proteins and fatty acids biosynthesis is the main factor governing the mentioned antagonism (Bhatia and Rabson, 1976; Rathke et al., 2005). Accordingly, Brennan et al. (2000) showed robust negative linear correlation between protein and oil content of rapeseed. The authors reported that the sum of proteins + oil was constant at ~62%.

In the current work, we studied the relations between N nutritional level and olive fruit oil content, first in a controlled experiment with trees grown in containers and then under field conditions. In the second phase, while trying to understand response mechanisms, we tested the protein – oil antagonism paradigm previously reported for oilseed crops (Holmes, 1980; Brennan et al., 2000).

## 2 Materials and methods

The study was conducted in two phases, the first - a controlled experiment, with trees grown in inert media in large containers (Gilat site, named “controlled” experiment henceforth) and the second was a six-year (2011-2016) field study in a commercial orchard (Negba site, named “field” experiment henceforth). In both, trees were exposed to a range of N fertilization levels and fruit oil content was assessed.

### 2.1 Controlled experiment

Olives were grown in containers at the Gilat Research Center, Israel (lat. 31°20´N, long. 34°39´E). Detailed experimental setup was described in Erel et al. (Erel et al., 2013a; Erel et al., 2013b). Briefly, two-year-old cv ‘Barnea’ olive plants were planted in Feb. 2006 in 60 l containers filled with type 4 (4-6 mm) granular perlite and positioned in the open air. Differential nutrient application treatments were initiated in September 2006 when trees reached ~1.5m height. The experiment used a randomized block design with six replicates. After the first harvest, in the autumn of 2007, the 60 l containers were too small to support further growth and thus, in December 2007, three replicates per treatment were transplanted to 500 l containers where they remained for two additional years (2008 and 2009). Nutrient solution was provided to trees twice a day at quantities set to provide 30% drainage by volume to avoid salt accumulation. Nutrient solution application and drainage volume, electrical conductivity (EC), pH and mineral concentrations were routinely monitored. N concentrations in the irrigation solutions are given in **Table 1**. P and K levels were uniform in all treatments at levels of 10 and 100 mg l^-1^, respectively.

**Table 1:** Nitrogen concentrations in the irrigation solution (average ± SD.).

Treatment	Solution N(mg l^-1^)		
N1	4 + 1.1		
N2	14 + 4.2		
N3	24 + 2.2		
N4	47 + 5.9		
N5	81 + 10.2		
N6	107 + 4.8		
N7	155 + 8.1		
N8	211 + 14.9		

There were eight N treatments at concentrations ranging from 4 to 211 mg l^-1^ N (**Table 1**). Nutrient solutions were prepared in containers containing a full regime of all additional nutrient elements (Erel et al., 2008). Concentrations of solution micronutrients are presented in **Table 2**. Solutions were prepared by dissolving, in 1.5 m^3^ containers, commercial grade salts in tap water from the local water supplier. The salts used were: KH_2_PO_4_, K_2_SO_4_, KNO_3_, NH_4_H_2_PO_4_, NaNO_3_, and NH_4_NO_3_. In all treatments, N was allocated as 90% $\text{N}{\text{O}}_{3}^{-}$ and 10% $\text{N}{\text{H}}_{4}^{+}$ .

**Table 2:** Nutrients concentrations in the irrigation solutions*.

Nutrient	K	P	Ca	Mg	S	B	Fe	Zn	Mn	Cu	Mo
Conc. Mg l^-1^	100.0	10.0	52.0	16.3	35.2	0.25	0.55	0.16	0.27	0.02	0.015

*Cl was supplied by the irrigation water, which had an average concentration of 70 mg l^-1^

The average fruit yield throughout the experiment is presented in **Table S1**.

### 2.2 Field experiment

The experiment was conducted in a commercial, intensive olive orchard, located in the southern coastal plain of Israel near Kibbutz Negba (31°39′7.50″N 34°40′54.00″E). A detailed description of the experimental setup is available in Haberman et al. (2019). The orchard was planted in 2007 with trees of cv. Barnea at distances of 4m x 7m, producing a density of 360 trees ha^-1^. Three hectares of the orchard were divided into plots, each plot consisting of at least 12 trees (three rows x four trees in a row). Two uniform trees in the center of each plot were selected for fruit sampling. The experimental set up was randomized blocks with seven plots per treatment level producing 14 replicate trees per treatment. Trees were irrigated twice a week through a drip irrigation system (one drip line per row of trees with 1.3 l h^-1^, drippers spaced every 0.75 cm) with fresh water (EC = 0.4-0.5 dS m^-1^). Weekly irrigation quantity was according to return of the potential evapotranspiration calculated by a modified Penman-Monteith equation (Allen et al., 1998) multiplied by a crop factor. Annual irrigation amounts varied from 480 to 620 mm. Average precipitation was 487 mm, occurring mainly in winter between October and April. Fertilizers were applied continuously through the irrigation system (fertigation), starting at the beginning of the irrigation season and during each irrigation event until annual target application levels were reached at the end of August. Before the initiation of the experiment (2007-2010), the orchard was fertigated with annual amounts of 250 kg N and 175 kg K ha^-1^, as commonly practiced in the region. The experiment began in June 2011 with the application of differential annual amounts of N: 0, 75, 150, and 300 kg N ha^-1^ season^-1^ (entitled: N0, N75, N150, and N300, respectively). The experiment duration was six consecutive years through 2016. After four years of no N application in treatment N0, severe N deficiency symptoms were visually observed, and therefore, in the 2015 and 2016 seasons, N fertilization of the N0 treatment was adjusted to 40 kg ha^-1^. Each fertilization treatment was individually controlled and monitored including treatment-specific valves and individually tailored and stored liquid fertilizer. The liquid fertilizers were prepared according to each treatment’s specification and supplied by ICL Israel. Each fertilizer combination was compiled such that annual application per hectare included, in addition to the N portion, 250 kg potassium (K), 35 kg phosphorous (P), 3.8 kg iron (Fe), 1.9 kg zinc (Zn), 940 g manganese (Mn), 140 g copper (Cu) and 100 g molybdenum (Mo). The ratios between nutrients were constant except the tested variable N, provided as 50% ammonium and 50% nitrate. Average K and P concentrations in the irrigation water were about 50 and 7 ppm, respectively. Nitrogen concentrations were about 8 (in the seasons 2015-2016), 15, 30 and 60 ppm for the N0, N75, N150 and N300 treatments, respectively. To validate nutrient concentrations and ensure accuracy, irrigation solution was sampled and analyzed from drippers every two weeks, from two plots of each N level.

The average fruit yield throughout the experiment is presented in **Table S2**.

### 2.3 Measurements

In both experiments, leaf analysis was routinely performed following the common sampling and diagnosing procedure (July, youngest fully developed leaf from non-bearing branches). The results were presented and discussed in our previous papers: for the controlled experiment: (Erel et al., 2013a; Erel et al., 2013b), and field experiment (Haberman et al., 2019). Generally, N concentration in the diagnostic leaf was very low in the low N treatments (1.2-0.6% in the controlled experiment and 1.2-1.3% in the field experiment) and high in the high N treatments (1.9-2.2% in the controlled experiment and ~1.8% in the field experiment). The tissue analysis confirms that the trees’ N status ranged from severe deficiency to luxurious N levels.

At the appropriate ripening stage, when ~50% of the fruits on a tree broke color to purple, all fruit were manually (controlled experiment) or mechanically (using trunk shaker, field experiment) collected. A representative sample of 2 kg fruit was collected from each tree. One kg subsample was crushed into paste using an Abencor (mc2 Ingenieria y Sistemas, Seville, Spain) crusher. Oil and water content in the fresh olive paste were determined using a near infra-red spectrometer (Olivescan, Foss, Denmark) (Zipori et al., 2016). Another subsample of ~50g olive paste was dried at 70^0^C to constant weight and milled into powder to determine N content. 100 mg of the powdered material was digested with sulfuric acid and hydrogen peroxide (Snell and Snell, 1950). N concentration was measured in the solution by an automated photometric analyzer (Gallery Plus 152, Thermo Scientific). Protein concentration in the fruit was calculated based on fruit N concentration multiplied by the conversion factor 6.25 as proposed by Mariotti et al. (2008).

In 2015, fruits were sampled at 10–30-day intervals, from early August to November. About one kg of fruit was handpicked from each individual tree (n =14). Since fruit load is a major determinant for oil accumulation and properties (Trentacoste et al., 2010; Bustan et al., 2014), after the harvest trees that had a fruit load lower than 14 kg tree^-1^ were discarded leaving 6-10 measured trees per N level. The sampled fruits were kept in a cool box in the dark until reaching the laboratory, where they were stored at -80^0^C. The pulp was separated from the endocarp, freeze-dried, and milled to powder. Metabolite analysis was carried out according to a modified method described by (Roessner-Tunali et al., 2003), where 100 mg of the powdered tissue was extracted in 700 μL cold methanol with 40 μL internal standard (ribitol, 0.2 mg in 1 mL of water). The samples were shaken for 20 min at 4 °C, centrifuged at 20,000 g, mixed with 750 μL HPLC grade water, vortexed, and centrifuged again. A 400 μL aliquot was dried by a SpeedVac (Alpha RVC, Christ), re-dissolved and derivatized for 90 min at 37 °C (in 40 μL of 20 mg mL^-1^ methoxyamine hydrochloride in pyridine) and treated for 30 min with 70 μL N-methyl-N-[trimethylsilyl]trifluoroacetamide at 37 °C and centrifuged. The metabolites were detected by a mass-spectrometer (Agilent 6850 GC/5795C, Agilent Technology) where one μL was injected in 1:50 split mode at 230 °C to a helium carrier gas at a flow rate of 1 mL min^−1^. Chromatography was performed by an HP-5MS capillary column (30 m × 0.250 mm × 0.25 μm) and the spectrum was scanned for m/z 50–550 at 2.4 Hz. Finally, the ion chromatograms and mass-spectra were evaluated by the Mass-Hunter software (Agilent) that identifies sugars and amino acids by comparison of retention times and mass-spectra with authentic standards (Sigma), while characterizing other metabolites through the NIST14 library. The concentrations of olive soluble sugars (glucose, fructose, mannitol, myo-inositol, and sucrose) were quantified using authentic standards (Sigma) calibration curves. The following sugar concentrations were used to generate the calibration curve (0, 16, 32, 64, 125, 250, 500, and 1000 µg ml^-1^ in water). Sugar calibration curve samples were analyzed as described above for the olive fruit pulp samples.

### 2.4 Data analysis

Oil content is presented on fresh (FW) and on dry weight (DW) bases. The first was measured directly (NIR), and the latter was calculated according to Eq. 1.


Eq. 1 Oil content (DW,%) = (oil content (FW,%)/(100−water content,%))*100

The composition of the three fruit phases: oil, water, and solids were calculated from the oil content (NIR) and water content (oven), with the solids portion calculated from the total fruit weight minus oil+water. Best fit regression equations were tested in JMP statistical software (ver. 16.0 SAS Institute Inc., Cary, NC) and the correlation coefficient and p-value are presented in the figures. To test the effect of fruit load on the interactions between fruit N and oil content an analysis of covariance (ANCOVA) model was built with fruit load as the covariance.

## 3 Results

### 3.1 Factors affecting fruit N concentration

**Figure 1:**
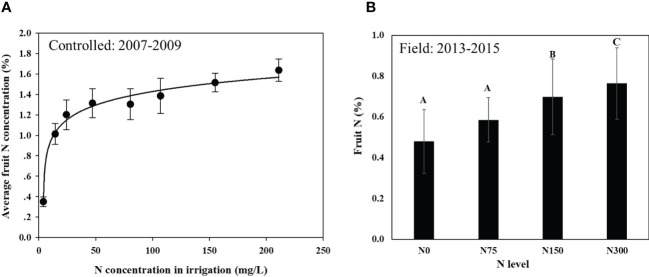
Average fruit N concentration in response to N application rate in the controlled experiment **(A)** and in the field experiment **(B)**. Data for the controlled experiment represent averages of three fruit-years with 6 replicates in the first year and 3 replicates in the following years (n=12) with a fitted logarithmic regression curve (R^2^ = 0.98; p<0.0001). Data for the field experiment are averages of the last three fruit-years (2013-2015) for14 replicates (n=42). Linear regression was fitted (fruit N = 0.512 + 0.0009181*N level), R^2^ = 0.90; p<0.05. Values labeled with different letters are significantly different following Tukey HSD test. * Is the regressions' equation.

Average fruit N concentration as a function of N concentration in the irrigation water is presented in **Figure 1**. Specific fruit N concentrations for a given year in the controlled container experiment was presented in Erel et al. (2013a). Fruit N increased sharply in the controlled experiment (**Figure 1A**) from 0.35 to 1.20% (more than a 4-fold increase) in response to increasing N levels from 4 to 24 mg l^-1^ (a 6-fold increase). When N levels increased further, from 24 to ~200 mg l^-1^ (about 8-fold increase) fruit N slightly increased from 1.20 to 1.64% at the highest N level. A logarithmic regression was found to best fit the response curve (p<0.0001). In the field experiment (**Figure 1B**), the fruit N values extended over a narrower range, from 0.48% N to 0.76%.

**Figure 2:**
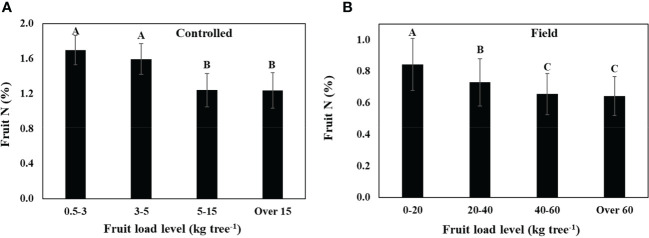
The effect of fruit load on fruit N concentration regardless of N nutritional level, **(A)** in young olive trees grown in large containers (controlled), and **(B)** in field-grown trees. Each category is an average of data collected during 2007-2009 in the controlled experiment (a, n=108), and 2011-2015 in the field experiment (b, n=126). Values labeled with different letters are significantly different following Tukey HSD test.

Low fruit load in trees receiving similar level of N was associated with an increase in fruit N both in trees grown in inert media in containers (controlled, **Figure 2A**), and under field conditions (**Figure 2B**). Further support for the existence of this correlation can be found in comparison between the two experiments: fruit load levels were much lower and fruit N was much higher in the controlled experiment compared to in the field, but the same trend prevailed in both experiments.

### 3.2 The association of fruit N with oil, water, and soils content

**Figure 3:**
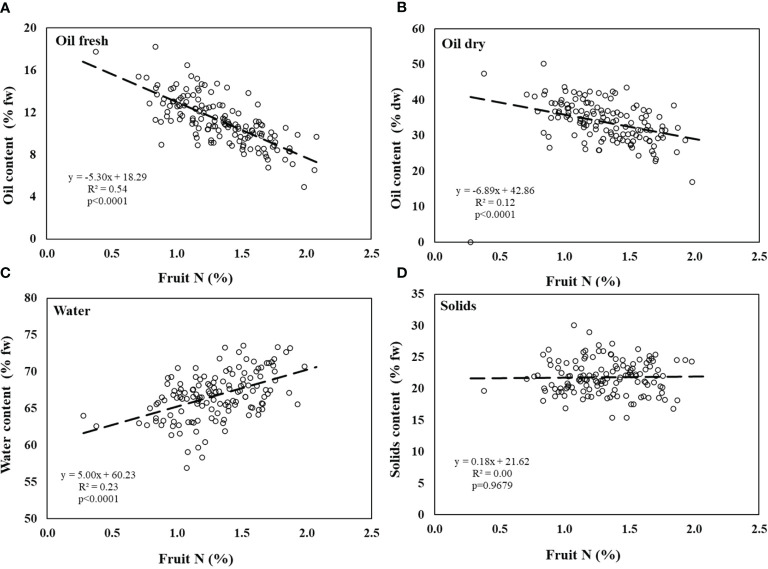
Olive fruit composition in the controlled experiment. **(A)** % oil by fresh weight, **(B)** % oil by dry weight, **(C)** water content, and **(D)** solids content as a function of fruit N concentration. Linear regressions of oil content (fresh), oil content (dry) and fruit water content are significant (p<0.0001). Solids content are not significantly affected by fruit N. Each symbol represents an individual tree at a given fruit year (2007-2009, n=96).

Results obtained from the controlled experiment show that there was a significant negative correlation between fruit N and oil content on a FW basis (**Figure 3A**). The same trend was observed between N in fruit and oil content on a DW basis, with more negative slope (-5.3 *vs.* -6.9) and lower R^2^ (0.54 *vs.* 0.12, **Figure 3B**). Water content was positively correlated to fruit N content (**Figure 3C**). The solid phase proportion (subtraction of water and oil from fruit weight) was not affected by fruit N concentration (**Figure 3D**).

**Figure 4:**
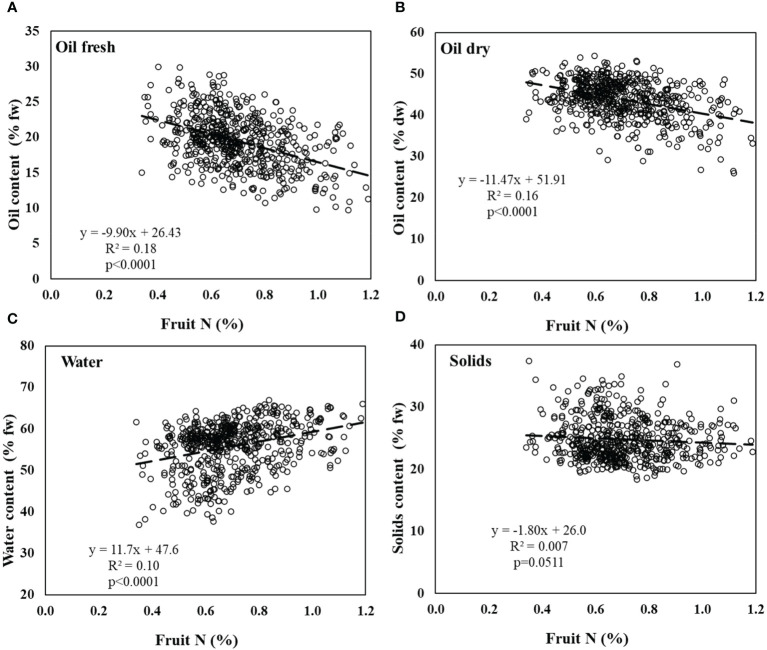
Olive fruit composition in the field experiment. **(A)** % oil by fresh weight, **(B)** % oil by dry weight, **(C)** water content, and **(D)** solids content in response to fruit N concentration. Linear regressions of oil content (fresh), oil content (dry) and fruit water content are significant (p<0.0001). Solids are not significantly affected by fruit N. Data was collected throughout the experiment, each point represents an individual tree at a given fruit year (2011-2015, n=570).

**Figure 5:**
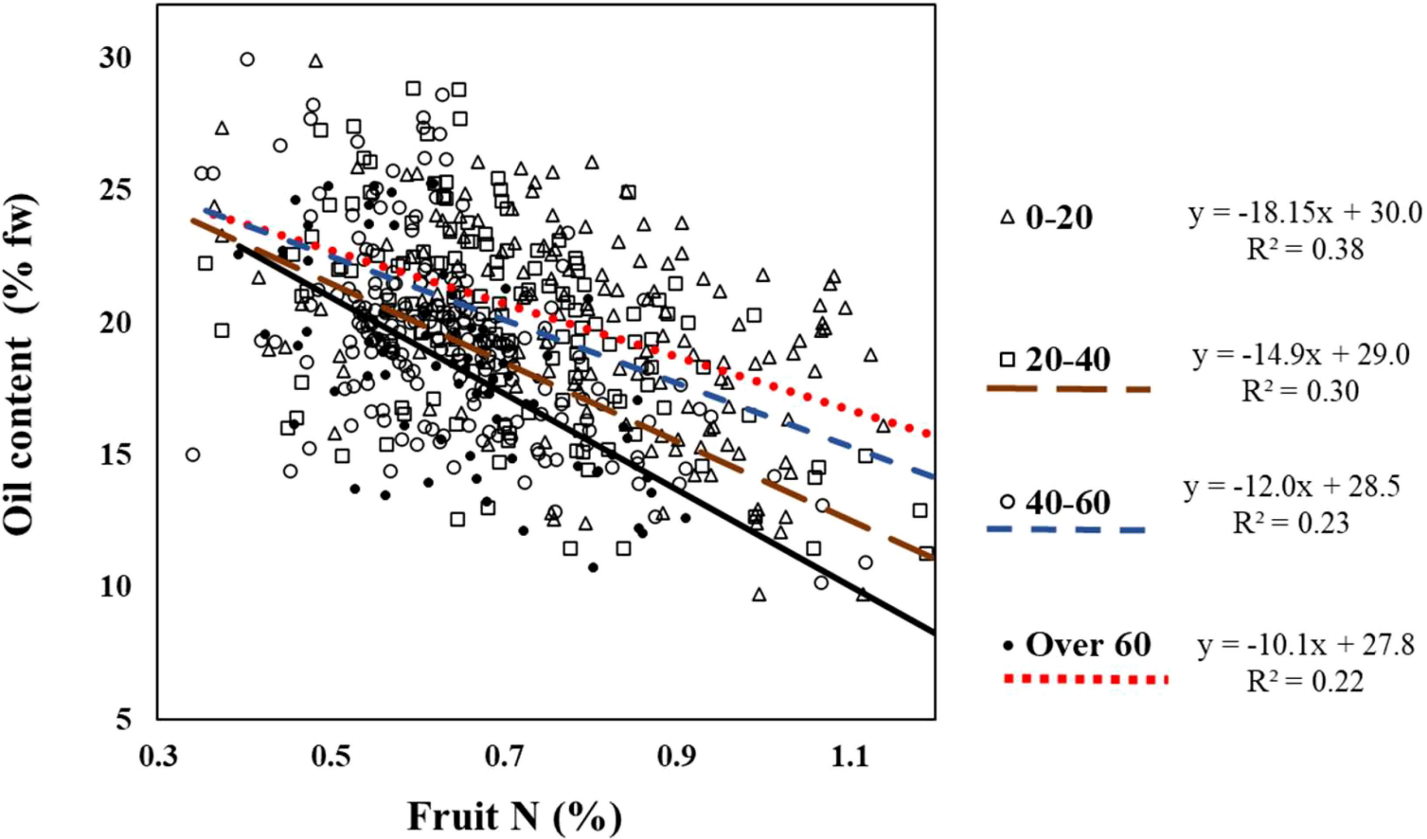
Relationship between fruit load category and the response of oil content to N in fruits from the field experiment. The interaction was tested using ANCOA model testing the response of oil content to fruit N for any given fruit load. Interaction is significant (p=0.0414) indicating fruit load had significant effect on fruit N – oil content interactions.

In the field experiment, there were more sampled trees than in the controlled experiment, but the N range in fruit was narrower, 0.4-2.1% N in the controlled experiment vs. 0.3-1.2% N in the field experiment. Yet, the same trends were observed; (1) a significant negative response of oil content on a FW basis to N in fruit (**Figure 4A**), (2) more negative slope in the response of oil content in fruit to N on a DW basis (**Figure 4B**), (3) a significant positive response of water content in fruit to N in fruit (**Figure 4C**), and (4) an absence of response of solid content (%) to N in fruit (**Figure 5D**).

### 3.3 Fruit load effect on the oil content - fruit N interaction

The slope of the regression line between fruit N and oil concentration increased with fruit load (i.e., -10.1, -12.0, -14.9 and -18.2, for low, medium, high, and very high fruit load respectively, **Figure 5**). This means that a higher fruit load led to a greater decline in oil content in response to fruit N.

**Figure 6:**
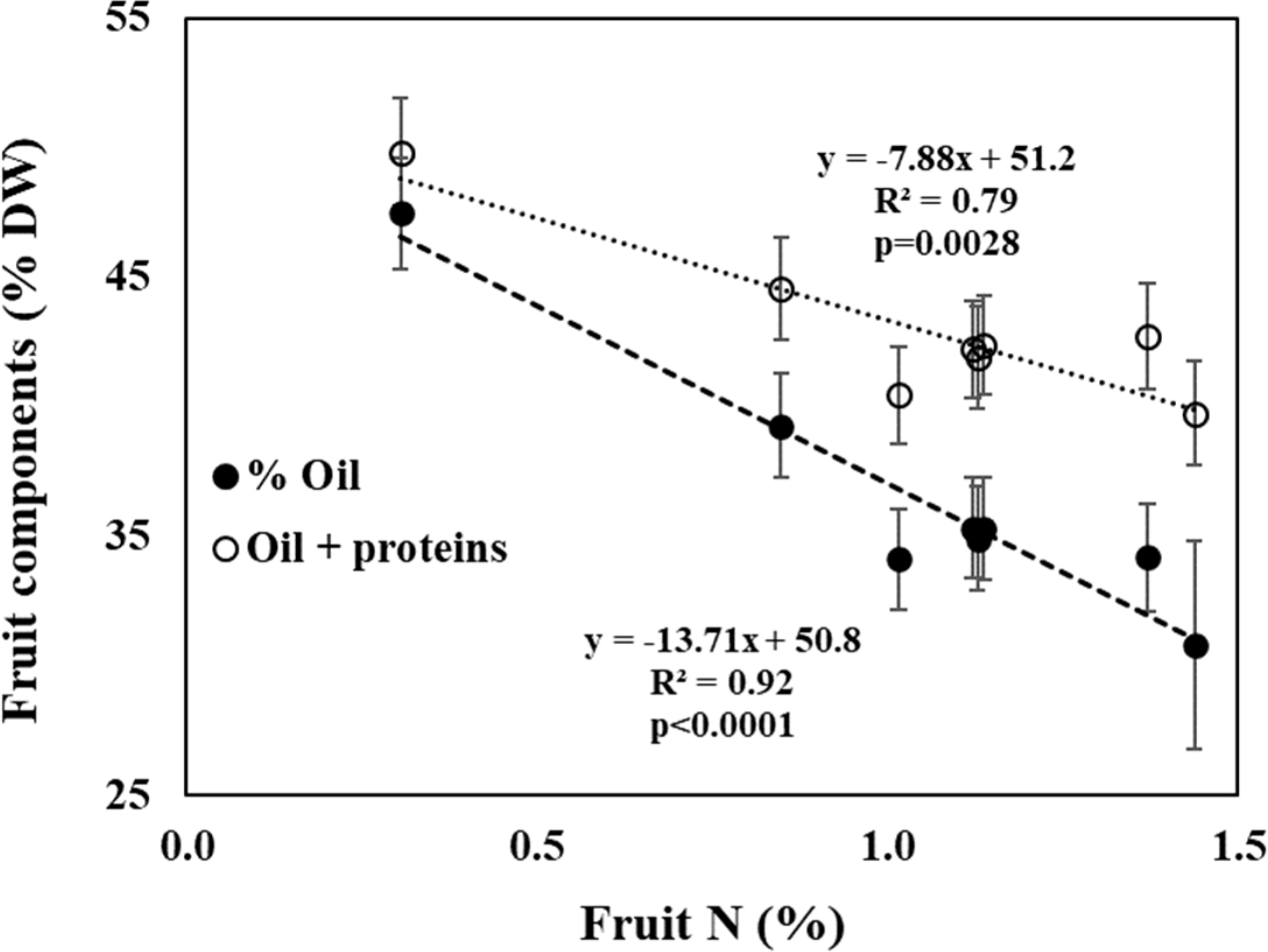
Fruit composition of oil (filled circles), oil + proteins (open circles) as a function of fruit-N concentration in the controlled experiment. Data represent averages ± SD of 2008 and 2009 (n=6).

The decline in oil content with fruit N can be merely a result of higher protein content in the fruit (i.e., dilution). If so, the sum of oil + protein should be constant. To test that, we analyzed the effect of N level on proteins + oil (**Figure 6**, controlled experiment; **Table 3** field experiment). The results indicate a significant decrease in oil + proteins with N level in both experiments. In the controlled experiment the portion of oil+proteins decreased from 49.8% to 39.8% and in the field experiment the portion of oil+proteins decreased from 49.4% to 45.1%.

**Table 3:** Fruit composition of oil, oil + proteins, and other solids as affected by N levels in the field experiment.

N level	Oil		Protein		Oil + proteins		Additional solids	
**N0**	46.1	a	3.3	b	49.4	a	50.6	b
**N75**	43	ab	4	b	47	ab	52.9	ab
**N150**	40	b	4.9	a	44.9	b	55.1	a
**N300**	39.7	b	5.4	a	45.1	b	54.8	a
**Linear**	***		***		**		**	
**Quadric**	***		***		**		**	

Each point is an average of 28 measured trees in 2014 and 2015. Linear and quadric regression were fitted, and p-value is presented below (*p<0.05; **p<0.001; ***p<0.0001). Values labeled with different letters indicate significant differences between treatments, following Tukey HSD test.

### 3.4 Accumulation of oil, N, and soluble carbohydrates during fruit development

**Figure 7:**
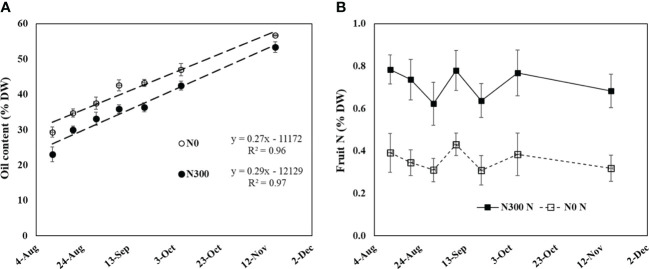
**(A)** Oil content and **(B)** fruit N content during fruit development in the field experiment (from August 11 to November 16, 2015) as affected by two extreme N fertilization doses. Each value represents an average of 6-10 samples. Error bars represent standard error.

On the last season on the field experiment (2015), frequent fruits sampling was done from early August to harvest to monitor N content, oil content and soluble sugars in the two extreme N levels. Oil content (based on dry weight) increased linearly over the period from 23% on August 11^th^ to 53.4% on November 16^th^ in fruit from the N300 treatment. Values were consistently higher in the N0 treatment, ranging from 29.4% to 56.7% in this period, maintaining a constant difference between the two treatments (**Figure 7A**). Nitrogen content in the fruit pulp was fairly constant during the period fruit maturation, with values for the N0 treatment being around 0.4-0.3% N and those for the N300 treatment more than double, around 0.7% (**Figure 7B**).

**Figure 8:**
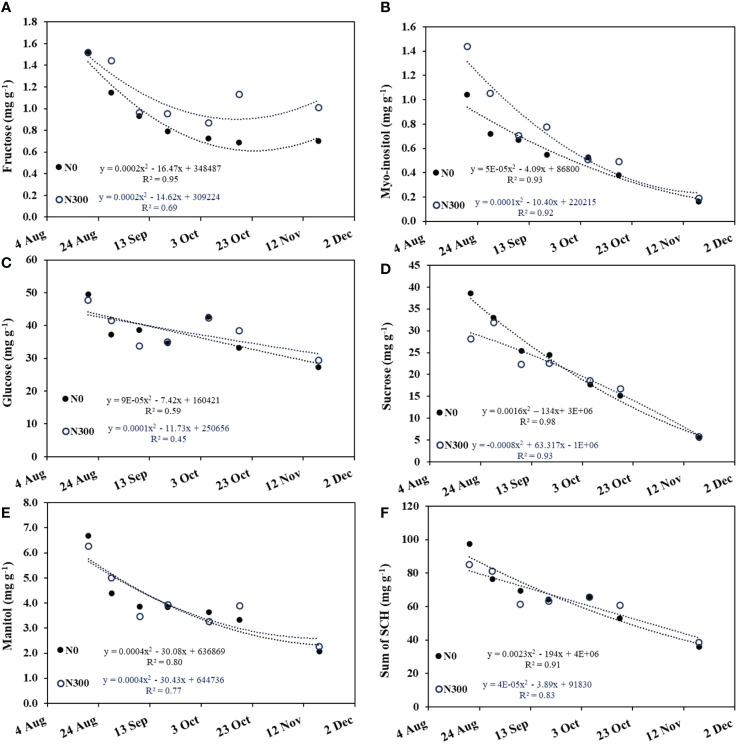
Changes in major soluble carbohydrate (SCH) concentrations in the fruit during the oil accumulation period (Aug. 20 to Nov. 18, 2015) at the two extreme N fertilization levels: N0 (filled circles) and N300 (open circles) in the field experiment. **(A)** fructose, **(B)** Myo-inositol, **(C)** glucose, **(D)** sucrose, **(E)** mannitol, and **(F)** sum of all major carbohydrates. Each datapoint is an average of 5-8 repetitions. Best fit regression (linear or quadric) is presented.

Glucose and sucrose were the dominant soluble carbohydrates (SCH) in the olive fruit, representing ~85% of the total. Major SCH concentrations decreased with time from the early stages of oil accumulation (August 20) until harvest (November 18), following either a polynomial decrease pattern in fructose, myo-inositol, and mannitol (**Figures 8A, B, E**) or a linear decrease in glucose and sucrose (**Figures 8C, D**). Overall, the total SCH showed pronounced linear decrease over time, regardless of N level (**Figure 8F**).

High N level was associated with increased concentrations of myo-inositol at the beginning of the season but not end of the oil accumulation period and increased total fructose over time. N fertilization level had little to no effect on the individual sugars glucose, sucrose, and mannitol (**Figures 8C-E**) and did not affect the concentrations of total soluble carbohydrates in the fruit pulp (**Figure 8F**; **Table 4**).

**Table 4:** Results of two-ways ANOVA testing the effect of N levels (categorical, 2 levels) time (continuous), and interaction on the concentrations of major soluble carbohydrates (SCH, **Figure 8**).

	Two-ways ANOVA					
	Fructose	Myo-inositol	Glucose	Sucrose	Mannitol	Total SCH
**Time**	***<0.0001***	***<0.0001***	***0.0014***	***<0.0001***	***<0.0001***	***<0.0001***
**N-level**	***0.0004***	***<0.0001***	0.9179	***0.0047***	0.6058	0.4198
**Interaction**	0.1452	0.0099	0.9006	***0.0103***	0.8293	0.2885

Significant effect is marked in bold italic.

## 4 Discussion

Oil accumulation rate, which results in the percentage of oil in olive fruit, has a major importance on orchard profitability as the oil yield is a multiplication of fruit yield by oil percentage. Previous studies have indicated various environmental aspects which influence olive oil accumulation: temperature (García-Inza et al., 2014; Nissim et al., 2020), salinity (Ben-Gal et al., 2017; Tietel et al., 2019), and water stress, especially in ‘On’ years (Ben-Gal et al., 2011; Naor et al., 2013). However, there are few reports on the effect of nutrient availability on oil accumulation in olives. Exposure to N is especially important since it is the main nutrient in olive fertilization management and is closely controlled by the grower. Here, we demonstrate, from both a container study and from a field experiment, that increased N availability resulted in increased N concentration in the fruit which, eventually, inhibited oil biosynthesis. Our results indicate that N level in fruit is a function of N fertilization level, fruit load, and their interactions (**Figures 3**-**5**).

A previous study from central Spain that explored the effect of N fertilization on productivity parameters in cultivars Arbequina and Picual olives under varied training/pruning strategies, did not find an effect of N treatment on oil percentage in olive fruit (Centeno et al., 2017). The inconsistency with the current study can be explained by the fact that the Centeno et al. (2017) study lasted two years, which likely was too short to obtain meaningful response under field conditions (Haberman et al., 2019). Fernández-Escobar et al. (2009a), in a long-term study focused on N fertilization, reported either no-effect or negative effect of N application level on fruit oil content. In that study in Spain, in 4 out of 10 years, there was a significant decrease in fruit oil content with increasing N application levels. Fernández-Escobar et al. (2009a) suggested that this was caused by a delay in fruit ripening induced by elevated N application level. However, their work was carried out in a rain-fed olive orchard while the current study was carried out under intensive, irrigated conditions.

High levels of N have been reported to adversely affect oil synthesis in various oilseed crops. Brennan et al. (2000) suggested that oil + protein concentration in rapeseed was almost fixed (around 62%), so that as one of them increased, the other would proportionally decrease. Similar findings were reported in additional studies on rapeseed (Holmes, 1980; Khan et al., 2018) and sesame (Golan et al., 2022). However, this phenomenon has not been reported so far in perennials and specifically olives, where the oil accumulates not in the seed but in the fruit pulp. In oilseed crops, the negative effects were explained by competition for assimilates between protein synthesis and oil synthesis (Brennan et al., 2000). While amino acids and protein synthesis are accelerated under high levels of N as a mechanism of the plant to store the excess N in the seeds, generally the sum of protein and oil concentrations is relatively constant for varying N fertilization levels (Brennan et al., 2000; Ma and Herath, 2016). Our results showed an increase in protein content in fruit under high N nutrition. However, the decrease in oil content was greater than the increase in protein suggesting that additional mechanisms were involved in the inhibition of oil synthesis under high N levels (**Figure 6**; **Table 3**).

In contrast to our initial hypothesis, the inhibition of oil synthesis was not found to be related to the SCH balance in the fruit, as most of its components were not affected by N fertilization levels throughout the oil accumulation period (**Figure 8**; **Table 4**). **Figure 8**; **Table 4** illustrate an increase in the fraction of total solids occurring with increasing fruit N concentrations. We suggest a complex physiological disorder caused by N over-fertilization (Sperling et al., 2019) as one possible explanation for the observed decrease in oil content in the high fruit-N range. At low N levels, lower vigor and hence sink strength can explain the increased availability of carbon for oil synthesis. Starch accumulation is a common response to nutritional deficiency (Paul and Driscoll, 1997) as shown previously for olives (Boussadia et al., 2010; Erel et al., 2016). If growth is restricted, more carbon might be available for oil synthesis (whereas N requirement is minimal), making oil the end point for assimilates.

Oil accumulation in cv. Barnea olives grown in Israel begins about 60 days after anthesis (Nissim et al., 2020). It seems that the negative impact of N nutrition on oil accumulation occurs during the early stages of fruit development, as from the beginning of August (about 150 days after anthesis) onward, the rate of oil accumulation was constant in both N0 and N300 treatments (**Figure 7**).

Our findings indicate a combination of two mechanisms in the inhibition of fruit oil accumulation in olives, caused by N over-fertilization. Excess N in the field experiment was previously reported to enhance vegetative growth (Haberman et al., 2019), suggesting whole-tree scale competition between vegetative and reproductive plant organs for photosynthates. Excess N finds its way to the fruit and competition occurs at the fruit level between two processes: oil accumulation and protein accumulation. When vegetative growth was impaired due to low N application rates, fruit oil accumulation was higher.

Various reports on the adverse effects of N over-fertilization on crop performance can be divided into two main categories: 1) Yield, including increased vegetative growth, reduced number of inflorescences, reduced flower bud initiation, and reduced fruit set (Albornoz, 2016) and 2) Quality, including reduced organoleptic and color attributes, reduced content of other mineral nutrients, reduced synthesis of secondary metabolites and increased nitrate content in the leaves (Albornoz, 2016; Zipori 2020). Specifically in olives, in addition to the reduction in oil synthesis as shown in the current study, N over-fertilization leads to a reduction in olive fruit yield associated with a reduction in flower quality (Fernandez-Escobar et al., 2008) and fruit set (Haberman et al., 2019). Also, N over-fertilization leads to a reduction in olive oil quality especially *via* elevated free fatty acid content, reduction in oleic acid content, reduction in polyphenol content and reduction in oxidative stability (Fernández-Escobar et al., 2006; Dag et al., 2009; Erel et al., 2013a; Pascual et al., 2019). Over-fertilization additionally increases environmental hazards as excess N, mainly as nitrate, leaches below the root zone creating contamination risks for deep soils and ground water (Puckett et al., 2011).

## 5 Conclusions

Olives are primarily cultivated for their excellent oil quality and beneficial health properties. Several agronomic practices, including fertilization, were shown to have a significant effect on oil production and properties. Here we show that N management has a central role in olive oil synthesis. In two independent experiments, fruit N level and oil biosynthesis were negatively associated. Fruit N level is affected by N fertilization and fruit load. The negative correlation between fruit N and oil content was more pronounced under high fruit load, indicating sink limitation for carbon. In opposition to reports on oilseeds, the paradigm trade-off between oil and protein cannot explain the noticeable decrease in oil biosynthesis in olives, indicating that additional mechanisms are involved in the N-induced inhibition of oil production. This inhibition was not related to the soluble carbohydrate levels in the fruit, which were comparable regardless of N level. The results highlight the importance of balanced N fertilization in olives cultivated for oil production.

## Data availability statement

The datasets generated for this study are available on request to the corresponding author.

## Author contributions

RE: conceptualization, methodology, formal analysis, investigation, writing. UY: conceptualization, methodology, investigation, funding acquisition, writing – review and editing. HY: metabolite analysis, writing – review and editing. AB-G: conceptualization, investigation, writing – review and editing. IZ: conceptualization, methodology, formal analysis, investigation, writing – review and editing. AD: conceptualization, investigation, writing – review and editing. All authors contributed to the article and approved the submitted version.
