# Supplementary material for: Elevated fruit nitrogen impairs oil biosynthesis in olive (Olea europaea L.)
Source: Front Plant Sci. 2023 Jun 30;14:1180391. doi: 10.3389/fpls.2023.1180391 (PMC10347680; doi:10.3389/fpls.2023.1180391)
Supplement: Supplementary file 1 [file DataSheet_1.pdf]

Table S1: Seasonal fruit yields (kg tree^{-1}) of the controlled experiment. Numbers are mean values of 6 replicates (trees) in 2007 and 3 replicates on 2008-2009 ± standard error of the mean.

N level	2007	2008	2009
**N1**	0.0 ± 0.0	0.8 ± 0.2	0.1 ± 0.0
**N2**	1.9 ± 0.2	10.2 ± 2.1	3.7 ± 0.6
**N3**	2.7 ± 0.4	16.2 ± 2.9	13.2 ± 1.8
**N4**	3.3 ± 0.4	25.9 ± 7.5	9.4 ± 4.3
**N5**	2.9 ± 0.5	15.9 ± 5.0	13.4 ± 3.8
**N6**	2.4 ± 0.6	18.0 ± 1.0	21.2 ± 1.5
**N7**	2.3 ± 0.5	10.7 ± 3.6	11.3 ± 3.2
**N8**	1.1 ± 0.2	5.8 ± 2.0	9.1 ± 1.7

Table S2: Seasonal fruit yields (kg ha^{-1}) of the field experiment. Numbers are mean values of 14 replicates (trees) ± standard error of the mean.

N level (kg ha^{-1})	2011	2012	2013	2014	2015	2016
**0,40***	16.9 ± 1.6	12.3 ± 1.5	12.8 ± 2.2	8.2 ± 2.2	6.2 ± 2.1	4.1 ± 1.3
**75**	13.8 ± 1.6	16.5 ± 1.5	10.8 ± 1.9	15.3 ± 2.2	5.1 ± 1.7	11.1 ± 1.6
**150**	15.4 ± 1.7	15.0 ± 1.2	15.1 ± 2.3	13.5 ± 2.3	9.0 ± 2.3	11.8 ± 1.9
**300**	16.8 ± 0.7	11.3 ± 2.0	15.9 ± 2.5	7.5 ± 2.3	10.7 ± 2.3	9.9 ± 1.9

* Plots did not receive N fertilization on 2011-2014 seasons, and on 2015-2016 seasons fertilized with an annual amount of 40 kg ha^{-1}.
